# Sexuality and Gender Role in Autism Spectrum Disorder: A Case Control Study

**DOI:** 10.1371/journal.pone.0087961

**Published:** 2014-01-31

**Authors:** Susanne Bejerot, Jonna M. Eriksson

**Affiliations:** Department of Clinical Neuroscience, Karolinska Institutet, Stockholm, Sweden; Lyon Neuroscience Research Center, France

## Abstract

The ‘extreme male brain theory of autism’ describes an extreme male pattern of cognitive traits defined as strong systemising abilities paired with empathising weaknesses in autism spectrum disorder. However, beyond these cognitive traits, clinical observations have suggested an ambiguous gender-typed pattern regarding several sexually dimorphic traits.

The aim of the present study was to investigate if patterns of non-cognitive sexually dimorphic traits differed between the autism spectrum disorder and control groups. Fifty adults with autism spectrum disorder and intelligence within the normal range, and 53 neurotypical controls responded to questions on gender role, self-perceived gender typicality and gender identity, as well as sexuality. Measures used were a Swedish modification of the Bem Sex Role Inventory and questions on sexuality and gender designed for the purpose of this study. Our results showed that one common gender role emerged in the autism spectrum disorder group. Masculinity (e.g. assertiveness, leadership and competitiveness) was weaker in the autism spectrum disorder group than in the controls, across men and women. Self-perceived gender typicality did not differ between the groups but tomboyism and bisexuality were overrepresented amongst women with autism spectrum disorder. Lower libido was reported amongst both male and female participants with autism spectrum disorder compared with controls. We conclude that the extreme male patterns of cognitive functions in the autistic brain do not seem to extend to gender role and sexuality. A gender-atypical pattern for these types of characteristics is suggested in autism spectrum disorder.

## Introduction

The extreme male brain theory of autism describes an autistic personality characterised by extremes of typical male personality traits [1] in terms of systemising skills and weaknesses in empathy [2], [3]. This connection to sex-related differences might be an important clue to the aetiology of autism spectrum disorder (ASD). Previous studies have shown that elevated levels of testosterone in amnion fluid predict autistic cognitive traits in childhood [4], [5]. In both sexes, prenatal exposure to testosterone affects the foetal brain resulting in masculinised future personality and behaviours [6], [7]. This is illustrated by an increased rate of tomboyism and masculinisation in women with congenital adrenal hyperplasia (CAH), an enzymatic defect resulting in highly elevated prenatal testosterone levels [8]. Although most women with CAH are heterosexual, rates of bisexual and homosexual orientation are elevated amongst this population [9]. Similarly, neural masculinisation [10] and tomboyism [11], [12] have also been reported in females with ASD. In addition, ASD has been shown to be overrepresented in men as well as women with gender identity disorder [13]. It has also been implied that gender identity disorder is overrepresented in ASD [14], [15]. Further, bisexuality and homosexuality are suggested to be more common in men with ASD than in men in the general population [16], while data on women with ASD is lacking.

Animal studies have indicated that abnormal prenatal testosterone levels may also affect sexual behaviour and desire in the adult animal [17], [18], but whether this relationship also applies to humans is unknown. Exposure to prenatal androgens seems to be one factor in the multifaceted aetiology of autism, in line with the extreme male brain theory of autism. A study of differences between ASD and typically developed individuals regarding other sexually dimorphic traits (presumably related to prenatal masculinisation or defeminisation) may add to the knowledge of the development of an androgen dependent form of ASD. To date, no studies have examined if the extreme male cognitive pattern and the lack of gender differences in systemising-empathising dimensions extend to other sexually dimorphic traits, such as gender role and sexuality in individuals with ASD. Clinical experience, however, suggests that masculinity, expressed as male typical territorial or sexual behaviour [19], [20], is attenuated in ASD of both sexes.

The current study constitutes the second half of a larger case control study on sexuality, androgen levels and anthropometric measures [21]. We found that the women with ASD had elevated testosterone levels and several physical masculinised characteristics, whereas the men with ASD displayed several physical feminised characteristics. However, the testosterone levels in the men with ASD did not differ significantly from the controls'. The current study further investigated how ASD relates to a set of non-physical attributes including gender role, gender identity, self-perceived gender typicality, androgynous behaviour in childhood, and sexuality. Gender role was assessed as adherence to a set of stereotypically male or female skills, while other aspects were self-reported. We hypothesized that male typical behaviour and perception would be weakened in ASD of both sexes, and that gender identity would be less pronounced than amongst controls.

## Methods

### Ethics statement

The study design was discussed in depth and approved by the local empowerment board within the Swedish Autism Society (Autism & Aspergerförbundet). The Regional Ethic committee in Stockholm approved the study protocol (Dnr 2005/644-31/3) and the investigation was conducted according to the principles expressed in the Declaration of Helsinki. All participants provided written consent and reimbursement was offered for participation (approximately £95). All participants who declined to participate or did not otherwise participate were not disadvantaged in any way by not participating in the study.

Individuals with ASD were recruited through a website for adults with ASD or through a requests sent to outpatients at a tertiary psychiatric unit for adults with ASD as well as to a community-based information centre. This centre offers information about legal aspects on the ASD diagnosis, social services etc., and is mainly serving adults diagnosed with ASD in teens or adulthood. The written request concerning the study included a brief information and the participant themselves either responded on e-mail or by post if they were interested in participating. Thereafter a brief telephone interview was conducted to ensure that the subject had attended mainstream schooling and had not been diagnosed with intellectual disability. The participants' ability to consent to the study was further confirmed in the interview by the first author, a senior psychiatrist with extensive training and experience with adult ASD. A total of three individuals were deemed not to have the capacity to consent and were subsequently excluded; two of these had a comorbid psychosis and the third had epilepsy and brain damage. None of the included participants had a caretaker or a guardian. Moreover, half of the ASD sample had a university degree and eight were parents themselves suggesting a relatively high level of functioning. For a capable group of adults with ASD – such as the participants in the present study – a requirement of consent from next of kin would presumably be perceived as humiliating, and the local empowerment board within the Swedish Autism Society did not support such a requirement.

### Participants and procedure

**Figure 1:**
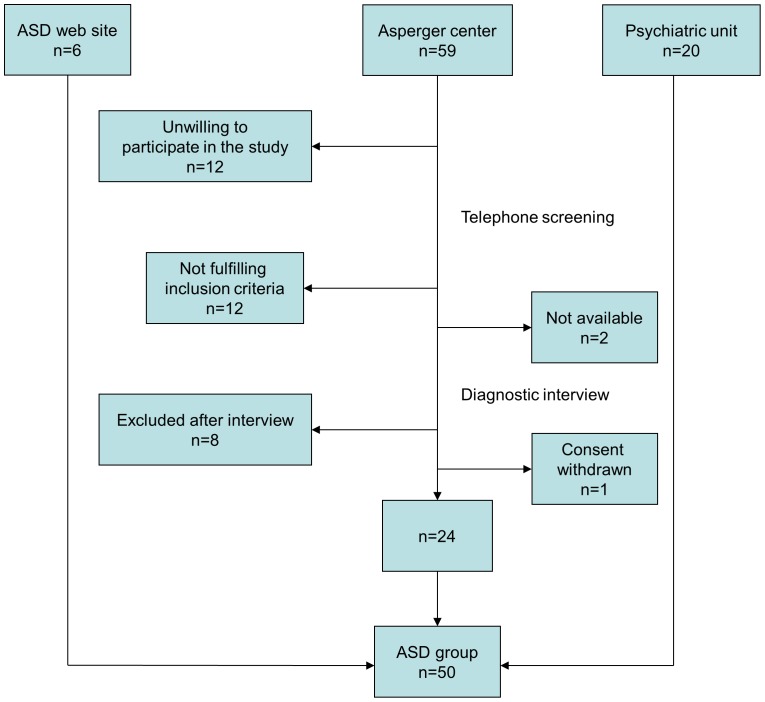
Flow chart for recruitment of participants with autism spectrum disorder.

The study involved 103 Swedish adults, including 50 adults (26 men, 24 women) diagnosed with ASD and 53 neurotypical controls (28 men, 25 women) matched on gender and age, demographics are shown in **Table** 1. Individuals with ASD were recruited through an outpatient tertiary psychiatric unit for adult ASD, a community-based centre for adults with ASD, and through a website for adults with ASD, see flowchart (**Figure** 1). Controls were recruited after the ASD group in order to be matched for gender and age. Sources for recruitment were a non-profit keep-fit organization, university, student residences, private companies, dentists and vaccination centres, employment agencies and through word-of-mouth recommendations. Inclusion criteria for both groups were Swedish/Caucasian descent and age between 20 and 47 years. Exclusion criteria were any disease or medication affecting androgen status, congenital syndrome, neurological or genetic syndrome, psychosis, diagnosed malformations, intellectual disability or having attended special education in primary or secondary school. Additional exclusion criteria in the control group were ASD or ASD in a first-degree family member. All participants denied any use of anabolic steroids and other androgen treatments other than hormonal contraceptives, used by 7 women with ASD and 14 women in the control group.

**Table 1:** Demographic data of the study samples.

		ASD group		Control group	
		Males (n = 26)	Females (n = 24)	Males (n = 28)	Females (n = 25)
Age, years: mean (s.d.)		31.8 (7.8)	28.1 (6.3)	32.9 (7.4)	27.7 (6.7)
Education, n					
	≤9 years	5	3	1	0
	≤12 years	9	9	2	5
	University level	12	12	25	20
Cohabiting with partner, *n* (%)		3 (11.5)	6 (25)	14 (50)	12 (48)
Having children, *n* (%)		5 (19.2)	3 (12.5)	8 (28.6)	3 (12.0)
The Autism-Spectrum Quotient: mean (s.d.)		28.0 (9.4)	31.9 (7.9)	11.7 (5.4)	10.4 (4.2)
Reading the Mind in the Eyes: mean (s.d.)		25 (5)	24 (4)	27 (3)	29.5 (2.5)
GAF, current (past month): mean (s.d.)					
	Symptoms	54 (13)	52 (12)	98 (4)	97 (5)
	Functioning	57 (13)	56.5 (13)	98 (3)	97 (5)

ASD; autism spectrum disorder.

Participants in the ASD group had all been diagnosed with ASD prior to this study and their medical records were reviewed. Local diagnostic procedures require that each patient is assessed rigorously by a psychiatrist and psychologist experienced with ASD over approximately 12–20 hours before receiving the diagnosis. The assessment includes neuropsychological tests, including Wechsler Adult Intelligence Scale, and interviews with parents to obtain a developmental history. For inclusion in the current study, a senior psychiatrist with extensive training and experience with adult ASD confirmed all diagnoses through an independent diagnostic interview using the Autism Diagnostic Observation Schedule [22] and also assessed general functioning [23]. Participants were interviewed for background information and gender typical behaviour in childhood. The questionnaires regarding gender role, gender identity and sexuality were completed by the participants.

### Assessment

#### Gender role

Gender role was measured using the MF scale [24], [25], a validated Swedish modification of the Bem Sex Role Inventory, measuring stereotypical masculine and feminine traits [26]. In contrast to the Bem Sex Role Inventory which presents a single adjective (e.g. ‘competitive’) the MF scale provide full statements (e.g. ‘I am competitive’). The MF scale consists of 43 items rated on a four-point Likert scale (1 = “I totally disagree”, 2 = “I slightly disagree”, 3 = “I slightly agree” and 4 = “I fully agree”). The male and female stereotypes (subscales labelled MF_M_ and MF_F_, respectively) are assessed with 17 items each, and a further 9 items are gender neutral. The MF_M_ subscale contains statements regarding power, assertiveness, leadership abilities and competitiveness, while the MF_F_ subscale mainly measures how tender, caring and submissive a person is. In order to estimate current masculine and feminine gender roles, we performed a pilot validation study of the MF scale in a large non-clinical Caucasian and Swedish born adult population sample (N = 637, 46% men and 54% women; age range 19–65 years) (un-published). This showed mean MF_M_ scores of 48.6 (6.3) for men and 45.3 (5.8) for women, and mean MF_F_ scores of 42.4 (4.8) for men and 46.4 (4.4) for women.

#### Gender perception, sexual debut and behaviours

Gender identity, androgynous behaviour, gender typicality and sexuality were examined through 10 self-rated items constructed for the purpose of this study. Each item had explicit wording and straightforward response alternatives. *Gender identity* was defined as to what gender the person feels he/she belongs to; *androgynous behaviour in childhood* was characterised as being sissy/tomboyish in childhood, and *gender typicality* was defined as how gender typical the person assessed him-/herself in adulthood, in relation to other people of the same sex. Regarding sexuality, we focused on areas that possibly – but certainly not exclusively – could be related to foetal testosterone levels: libido, taking sexual initiatives, sexual interest, orgasms and sexual orientation [8], [27]. Additionally two items on sexual debut were included. The items and their response options are presented in **Table 2**.

**Table 2:** Gender perception, sexual debut and behaviour items.

Measure	Item	Response alternatives	Dichotomized responses	Interpretation
***Gender perception***				**as a gender-atypical pattern**
Gender identity	What is your gender identity?	“Woman”, “In-between man and woman”, “man”, “transsexual”		
			In between man and woman, transsexual, opposite sex	Yes
			Biological sex	No
Androgynous behaviour in childhood	Were you a ‘sissy’/tomboy during childhood?	“Yes”/“No”/“Don't know”		
			Yes	Yes
			No	No
Gender typicality	Do you perceive yourself as typical for your gender?	“Yes, absolutely” “Yes, to some extent” “No, not at all” “Don't know”		Reversed item, i.e. “No” indicates gender atypicality and “Yes, to some extent” some gender atypicality.
***Sexual debut***				
Intercourse	Have you had sexual intercourse?	“Yes”/“No”	Yes/No	
Debut age	Age for sexual debut?	Age in years		
***Sexual behaviour***				**as a masculinised pattern**
Libido	Have you been sexually aroused past month?	“No”, “1–3 times”, “4–6 times”, “7–15 times”, “every day”		
			Higher ≥4 times	Yes
			Lower <4 times	No
Sexual initiative	Who takes initiative to have sex?	“I”, “both”, “partner”, “no one”		
			Initiator: I, both	Yes
			Not initiator: partner, no one	No
Sexual interest	Are you interested in sex?	“No, not at all”, “Not very much”, “Yes, quite”, “very interested”		
			“Yes, quite”, “very interested”	Yes
			“No, not at all”, “Not very much	No
Orgasm frequency	Have you had an orgasm during the past month?	“No”, “1–3 times”, “4–6 times”, “7–15 times”, “every day”		
			Higher ≥4 times	Yes
			Lower <4 times	No
Sexual orientation	Whom are you attracted to?	“Men”, “women”, “both men and women”, “neither”, “other”		
			Attracted to females	Yes
			Not attracted to females	No

Note. Response alternatives “don't know” were treated as missing data.

#### Measures of cognitive autistic traits and functioning

To describe the groups, two measures of cognitive traits, commonly used in ASD evaluations were included, The Autism Spectrum Quotient (AQ) [28] and “The Reading the Mind in the Eyes test” [29]. The AQ is a 50 item self-evaluation questionnaire, assessing personal preferences and habits, and “The Reading the Mind in the Eyes test” is a measure of mentalising skills through testing the ability to decipher emotions in photos of expressive sets of eyes. Both instruments have shown gender-variations in neurotypical people. General functioning was quantified using the Global Assessment of Functioning (GAF) [23]. Symptoms and functioning are assessed separately, each with scores ranging from 0 to 100.

### Statistical analysis

Comparisons between participants with ASD and controls were made using Student's t-test for normally distributed data, the Mann-Whitney U test for non-parametrical data and χ^2^-test for categorical variables. Missing data on the MF_M_ and MF_F_ subscales (0.06%) and the AQ (0.4%) was substituted using the mean value of all the items in the respective scale. A two tailed P value of <0.05 was considered significant. The gender identity was dichotomised into either biological sex or not (in-between man and woman, transsexual and non-biological sex). Sexual behaviour items were dichotomised for more straight forward interpretation of masculine dimensions of sexuality (**Table** 2).

## Results

### Gender role, gender identity and androgynous behaviour in childhood

As shown in **Table** 3, both men and women with ASD rated themselves as having a less masculine gender role than the controls, according to the MF_M_ subscale. More individuals with ASD than controls reported an atypical gender identity (χ^2^(1, N = 103) = 10.1, φ = 0.31, P = 0.001). When separated by sex, this reached significance only for the woman. Although no difference was found between men with ASD and controls on being ‘a sissy in childhood’, women with ASD rated themselves as being more tomboyish in childhood compared with the female controls. However, regarding self-perceived gender typicality, no significant difference was shown between the ASD group and controls for either sex (Men: MWU = 335, P = 0.98; Women: MWU = 235, P = 0.34). **Table** 4 shows the correlations between the gender role and gender perception measures in ASD males and females respectively. Additional correlations as well as frequencies and percentages of non-dichotomised gender perception data are presented in **Table** S1–S5 in File S1.

**Table 3:** Gender role and gender identity measures in 50 individuals with autism spectrum disorder (ASD) and 53 neurotypical controls.

		ASD group			Control group					Effect size	
*MF Gender role*	Sex	N	Mean(s.d.)	z-score	N	Mean(s.d.)	z-score	t(df)	P	r	
MF_M_ subscale score	M	26	41.7(6.2)	−1.09 (0.99)	28	47.9(6.0)	−0.1 (0.96)	−3.7(52)	0.0005	0.46	↓
	F	24	40.0(6.6)	−0.91 (1.13)	25	47.2(5.8)	0.33 (0.99)	−4.1(47)	0.0002	0.51	↓
MF_F_ subscale score	M	26	44.6(6.1)	0.45 (1.28)	28	42.2(4.2)	−0.04 (0.87)	1.7(52)	0.1	0.22	±
	F	24	45.8(6.2)	−0.15 (1.41)	25	46.9(4.3)	0.11 (0.98)	−0.8(47)	0.4	0.11	±
*Gender identity and gender behaviour*			*n*(%)			*n*(%)		χ^2^(df)	P	Φ	
Gender-atypical identity in adulthood	M	26	3(11.5)		28	1(3.6)		1.3(1)	0.3		±
	F	24	8(33)		25	0(0)		10.0(2)	0.002	0.45	↑
†Androgynous behaviour in childhood	M	22	5(23)		28	7(25)		0.04(1)	0.8	-	±
	F	19	12(67)		24	8(33)		3.9(1)	0.05	0.31	↑

MF_M_ and MF_F_ scales' z-scores normalised for gender by data from the MF validation study. ↑ denotes an increase and ↓ a decrease in masculinity.

M = Males; F = Females;

MF_M_ = The Masculine subscale score for self-rated masculine gender role;

MF_F_ = The Feminine subscale score for self-rated feminine gender role; MWU = Mann-Whitney U test.

† 2 men and 4 women with ASD and 1 woman in the control group responded “I don't know” and thus excluded in the analyses; in addition to missing data in 2 men and 1 woman with ASD.

**Table 4:** Spearman correlations of gender perception measures in autism spectrum disorder.

	Gender identity	Androgynous in childhood	Gender typicality	MF_M_	MF_F_
Gender identity	-	0.10	0.35	−0.41*	0.49*
Androgynous in childhood	0.13	-	0	−0.04	0.23
Gender typicality	0.43*	0.22	-	−0.47*	0.06
MF_M_	0.19	0.22	0.37	-	−0.08
MF_F_	−0.43*	−0.17	−0.03	0.03	-

Showing the men above the diagonal and the women below the diagonal.

Note. Due to missing data N varies between 19 and 26.

* P<0.05.

### Sexuality measures

**Figure 2:**
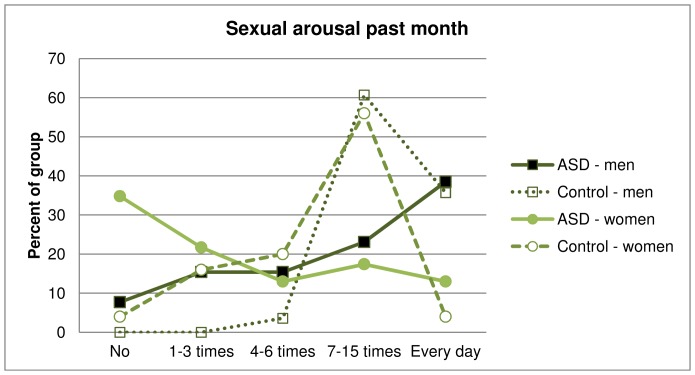
Frequencies of sexual arousal in men and women with and without autism spectrum disorder.

Results for the sexuality measures are presented in **Table 5** and correlations between the measures in **Table 6**. Four men and four women (16%) in the ASD group had never had intercourse at the time of the interview, significantly more than reported by the controls (χ^2^ = 9.39; P = 0.002). Amongst the participants that had experienced their sexual debut, this occurred later for the men-women combined ASD group (mean age: ASD males = 22.1(5.9), ASD females = 18.7(4.7); Control males = 17.4(4), Control females = 16.5(2.7); MWU_combined_ = 844, N_ASD_ = 43, N_C_ = 53, P = 0.03, r = 0.13). The combined ASD group reported fewer moments of sexual arousal (χ^2^(1, N = 102) = 12.0, P = 0.0005, φ = 0.34), less sexual interest (χ^2^(1, N = 103) = 10.0, P = 0.002, φ = 0.31) and less inclination to initiate sex compared with the controls (χ^2^(1, N = 95) = 18.0, P = 0.00002, φ = 0.44). **Figure** 2 shows frequencies of sexual arousal in men and women with and without ASD. Fewer men and women with ASD fell into the high frequency of orgasms group (≥4 times monthly). Notably, ten women (44%) with ASD had not had any orgasm the previous month, compared to 4 women (16%) in the control group. Homosexuality or bisexuality were equally common in the male groups (homosexual, *n*_ASD_ = 2; *n*_C_ = 2; bisexual, *n*_ASD_ = 1; *n*_C_ = 2). However, amongst the women, sexual attraction towards other females was reported more frequently in the ASD group (homosexual, *n*_ASD_ = 1; *n*_C_ = 0; bisexual, *n*_ASD_ = 13; *n*_C_ = 4). One woman and one man with ASD reported asexuality. Frequencies and percentages of non-dichotomised sexuality data as well as correlations within the male and female ASD groups respectively are presented in **Table** S6–S11 in File S2.

**Table 5:** Sexuality parameters; ↑ denotes an increase and ↓ a decrease in masculine sexuality in autism spectrum disorder (ASD) compared with controls.

			ASD group		Control group			Effect size	
	Sex	N	*n* (%)	N	*n* (%)	χ^2^(df)	P	Φ	
Sexual intercourse	M	26	22(85)	28	28(100)	4.6(1)	0.05	0.29	↓
	F	23	19(83)	25	25(100)	4.7(1)	0.05	0.31	↓
Libido	M	26	20(77)	28	28(100)	7.3(1)	0.007	0.37	↓
	F	22	13(59)	25	20(80)	6.8(1)	0.009	0.38	↓
Sexual initiatives	M	22	15(68)	28	28(100)	4.6(1)	0.05	0.29	↓
	F	19	9(47)	25	23(92)	8.9(1)	0.005	0.45	↓
Sexual interest	M	26	20(77)	28	25(89)	1.5(1)	0.3	0.17	±
	F	24	11(46)	25	22(88)	9.9(1)	0.002	0.45	↓
Orgasm frequency ≥4 times monthly	M	26	17(65)	28	25(89)	4.4 (1)	0.03	0.29	↓
	F	23	7(30)	25	15(60)	4.2 (1)	0.04	0.30	↓
Sexual attraction towards females or both males and females	M	25	23(92)	28	26(93)	1.1 (2)	0.6	0.14	±
	F	22	14(64)	25	4(16)	11.2 (2)	0.0008	0.49	↑

M = Males; F = Females; Libido dichotomised into sexual arousal ≥4 times monthly (y/n), sexual initiative were dichotomized into ‘takes initiative’ (y/n); sexual interest (y/n); orgasm dichotomised into ≥4 times monthly (y/n).

**Table 6:** Spearman correlations of sexual measures.

	Libido	Sexual initiative	Sexual interest	Orgasm frequency
Libido	-	0.228	0.603***	0.718***
Sexual initiative	0.171	-	0.452**	0.249
Sexual interest	0.347*	−0.091	-	0.440**
Orgasm frequency	0.629***	0.295*	0.329*	-

Showing the autism spectrum disorder group above the diagonal and control group below.

Note. Due to missing data N varies between 42 and 53.

* P<0.05,

** P<0.01,

*** P<0.001.

## Discussion

In this study we have compared a set of sexually dimorphic characteristics between men and women with ASD and age matched controls. Differences in gender role, gender identity, gender behaviour in childhood and sexual behaviour and orientation were observed between individuals with ASD and neurotypical controls. Furthermore, gender differences across almost all measures were less pronounced in the ASD group than in the control group.

As foetal exposure to androgens also affects other aspects of the adult personality such as gender role, gender identity, gender behaviour and sexual orientation [30], it is reasonable to expect these traits to be similarly affected, that is, masculinised in women with ASD. This effect was partly supported in the present study. Women with ASD reported gender behaviour in childhood, adult gender identity and sexual orientation towards a masculine profile, whereas no significant differences in these characteristics were observed amongst the men. In contrast however, other measures pointed towards a de-masculinisation across gender. Both men and women with ASD reported an almost a-masculine gender role. Compared with controls, they reported lower libido, less likelihood to take sexual initiatives in romantic relationships and lower frequencies of both sexual arousal and orgasms. Although asexuality was reported in ASD, it was not reported amongst the controls. Taken together this suggests a reduced (or absent) sexual drive and de-masculinised sexual behaviour amongst the ASD participants. These findings align with the observations of attenuated masculine physical features previously reported in the ASD men, but contrast to the enhanced androgynous physical features (and elevated testosterone levels) observed in the ASD women [21].

### Gender role

The ASD group reported to have a gender role characterised by weak masculinity according to the MF scale. Intriguingly, for both men and women, the control group and the validation study sample reported higher scores on masculine gender role scale than both women and men with ASD. Moreover, the gender role in ASD participants was similar for men and women. An absence of sex differences in cognition regarding empathising and systemising skills which typically are dimorphic for sex, has been previously reported in ASD [1], [2]. Similarly, the current findings suggest a gender role independent of sex in ASD – a gender defiant gender role.

### Gender identity and typicality

Contradictory to the non-masculine gender role, ASD women reported greater masculinised gender behaviour in childhood as well as masculinised adult gender identity than control women. Two thirds stated that they were tomboys in childhood, compared with only one third of the female controls. The finding is congruent with earlier reports of increased tomboyism in female ASD [11], [12]. Although the men with ASD did not differ from the male controls in regard to gender identity or gender behaviour in childhood, a genetic male diagnosed with gender identity disorder and ASD was initially included in this study, but withdrew (data not included). Another participant with ASD, genetic female, was similarly diagnosed with gender identity disorder. ASD is suggested to be overrepresented amongst people with gender identity disorder of both sexes [13]. In addition, a study of people with gender identity disorder found that the genetic females (but not the males) reported high scores on the AQ [31]. Only the genetic males that were either asexual or heterosexual in relation to birth sex scored similarly as high as the genetic females on the AQ [32]. Consistent with those findings, the three participants in the current study with asexuality or gender identity disorder scored above the median on the AQ. Whether asexuality, gender identity disorder and ASD share similar pathophysiology should be further investigated.

Self-perceived gender typicality did not differ significantly between the ASD females and the female controls. However, as gender typicality and gender identity correlated in ASD females, it is plausible that an effect would emerge in a larger sample.

### Delayed sexual debut

In the current study the ASD group was less prone to initiate sex in a romantic relationship than the controls. Moreover 16% in the ASD group had never experienced intercourse. People with ASD are typically unsuccessful in forming social relationships and the majority are victimised by peers in childhood [33]. Plausibly, a history of rejection will result in low self-esteem and social withdrawal. In order to approach another person with sexual intent, initiative, as well as assertiveness and flirting abilities are critical; abilities that are poorly developed in ASD. As a result, sexual debut may be postponed or never occur.

### Libido

Another reason for a delayed sexual debut could be low libido. In this study the participants with ASD reported a lower libido and orgasm frequency than the controls. In addition, asexuality was reported by two of the ASD participants. Several reports by people with ASD suggest that asexuality is not rare in this population [15], [34], [35]. The individual's level of sexual desire is the result of a complex interplay between many physiological and psychosocial factors [36]. Although prenatal levels of androgens affect sexual orientation and behaviours throughout life, the relationship to sexual drive in adults is as yet unknown. Adult levels of sex hormones however, do play an important role in sexuality, but variation in testosterone levels within normal ranges has not been shown to correlate with level of sexual desire [37]. Thus, the low libido reported by our participants with ASD cannot be explained by low testosterone levels as these were not lower than in the controls [21].

### Sexual preferences

Although homosexuality was equally common in ASD and controls, bisexuality was acknowledged four times more frequently amongst women with ASD than amongst female controls. In the male ASD group, only one reported bisexuality compared with two amongst the male controls. Our sample was too small to reliably identify any small variations in sexual orientation. However the observed prevalence for the control men (14%) and for the ASD men (11%) both seem high, compared with estimates in British and American populations (4.6%, 5.4%) [38], [39]. Although our results neither support, nor oppose the hypothesis of an increased rate of homo- and bisexuality in men with ASD, other studies lend support for this hypothesis. Aston interviewed 28 men with Asperger syndrome who previously or currently lived with a woman [40]. Although they stated to be heterosexual, three of them reported sexual relationships with other men. In another cohort of 24 men with ASD living in residential care, 17% were bisexual or homosexual [16]. Taken together, this suggests an elevated rate of homo- and bisexuality amongst the male ASD population similar to the female.

There are a number of possible explanations for increased bisexuality in ASD. Bisexuality could reflect independence towards social norms in the society, a standpoint that is common in the ASD population. Another plausible explanation is “gender blindness” that leads to an appraisal of a potential partner's qualities rather than the persons' specific gender. Alternatively, increased rates of bisexuality and male-type behaviour in women could be a delayed consequence of elevated levels of androgens in foetal life.

### Limitations

A study of the current size addressing sexuality is likely to be affected by selection bias. Compared to an age equivalent clinical sample of adults with ASD [41], parenthood and university education were more common in the present study, but equivalent to participants in a study on sexual wellbeing in ASD [42]. This suggests that the presently studied ASD group was relatively resourceful and could possibly be considered more “neurotypical” than ASD subjects in general. Conversely, the male controls reported higher rates of non-heterosexuality than what could be expected in the general population and 25% stated being a sissy in childhood. In sum, our male controls are presumably less gender typical than the general population, which in fact strengthens our hypothesis on ASD as being a gender-atypical disorder. Furthermore, this may explain the non-significant findings in some of the analyses.

More women in the control group than in the ASD group used contraceptive pills. Although contraceptive pills may affect sexual interest, orgasm frequency and libido there are no conclusive data on which direction these functions are affected [43]. Also, fewer participants with ASD than controls had a partner. In this study, which also included assessment of hormonal and physical measures, we only matched the participants for age, sex and absence of tattoos in order to avoid selection bias. In a future study a preferable option would be to match controls for partnership and hormonal contraception.

For this particular study we constructed self-report items on gender behaviour in childhood, gender identity and sexuality in adulthood (Table 2). The use of a non-validated instrument could be viewed as a limitation but this was a necessary procedure in order to cover areas of interest, while keeping the number of items to a minimum. Long questionnaires have resulted in high dropout rates when applied to a comparable ASD population [42], something we wanted to avoid. It would have been preferable for a parent to confirm gender role behaviour in childhood. However, a prerequisite for a parent interview may create selection bias by excluding adults who reject parental interference (this applies to both subjects with ASD and controls). The validity of our findings is supported by the fact that responses on gender role and sexuality both suggested a weak masculine gender role and low libido in ASD. In addition, a number of case reports provide testimonies on gender-atypical life styles in the ASD population [15], [44]–[49]. However, considering the small sample size in this study, current tests need replication in a larger sample.

## Conclusion

This study lends support to a de-masculinised gender role independent of sex in the ASD population. The typical gender role characteristics associated with masculinised sexuality are rarely expressed in ASD, nor are ASD groups particularly feminine, with tomboyism common among women. People with ASD appear to have a lower sexual drive and minority sexual status is overrepresented compared with the non-clinical population.

While exhibiting an extreme male pattern of systemising and empathising skills across gender, men and women with ASD display a similarly gender defiant, however androgynous, gender identity. Concerning sexual behaviours and gender role a conspicuously de-masculinised pattern emerged. The extreme male brain theory of autism makes no claims about masculinity, thus not contradicting our findings. Whether prenatal androgen levels or other factors cause gender atypicality remain to be studied.

## Supporting Information

File S1 Contains Tables S1–S5.
(DOC)

File S2 Contains Tables S6–S11.
(DOCX)
