# Supplementary material for: Sexuality and Gender Role in Autism Spectrum Disorder: A Case Control Study
Source: PLoS One. 2014 Jan 31;9(1):e87961. doi: 10.1371/journal.pone.0087961 (PMC3909328; doi:10.1371/journal.pone.0087961)
Supplement: File S1 — Contains Tables S1–S5. (DOC) [file pone.0087961.s001.doc]

**Table S1 Gender identity.** What is your gender identity?

| Gender identity | N (%) | | | |
| --- | --- | --- | --- | --- |
|  | Men | | Women | |
|  | ASD | Control | ASD | Control |
| Ok with gender | 23 (88.5) | 27 (96.4) | 16 (66.7) | 25 (100) |
| In-between | 3 (11.5) | 1 (3.6) | 7 (29.2) | 0 (0) |
| Cross gender | 0 (0) | 0 (0) | 1 (4.2) | 0 (0) |
|  | 26 | 28 | 24 | 25 |

**Table S2 Androgynous in childhood.** Were you a ‘sissy’/tomboy during childhood?

| Androgynous in childhood | N (%) | | | |
| --- | --- | --- | --- | --- |
|  | Men | | Women | |
|  | ASD | Control | ASD | Control |
| Yes | 5 (20.8) | 7 (25) | 11 (50) | 8 (32) |
| No | 17 (70.8) | 21 (75) | 7 (31.8) | 16 (64) |
| Don't know | 2 (8.3) | 0 (0) | 4 (18.2) | 1 (4) |
|  | 24 | 28 | 22 | 25 |

**Table S3 Self perceived gender typicality.** Do you perceive yourself as typical for you gender?

| Gender typicality | N (%) | | | |
| --- | --- | --- | --- | --- |
|  | Men | | Women | |
|  | ASD | Control | ASD | Control |
| Yes, absolutely | 11 (42.3) | 12 (42.9) | 8 (33.3) | 10 (40) |
| Yes, quite | 10 (38.5) | 14 (50) | 10 (41.7) | 15 (60) |
| No | 3 (11.5) | 2 (7.1) | 4 (16.7) | 0 (0) |
| Don't know | 2 (7.7) | 0 (0) | 2 (8.3) | 0 (0) |
|  | 26 | 28 | 24 | 25 |

Table S4 Spearman correlations of gender perception and gender role measures in the combined male groups above the diagonal and female groups below.

| All Men | **Gender identity** | **Androgynous in childhood** | **Gender typicality** | **MF_M_** | **MF_F_** |
| --- | --- | --- | --- | --- | --- |
| **Gender identity** |  | 0.007 | 0.34^*^ | -0.33^*^ | 0.28^*^ |
| **Androgynous in childhood** | 0.18 |  | 0.13 | 0.09 | 0.05 |
| **Gender typicality** | 0.35^*^ | 0.32^*^ |  | -0.42^**^ | 0.15 |
| **MF_M_** | -0.15 | -0.07 | 0.04 |  | -0.30^*^ |
| **MF_F_** | -0.33^*^ | -0.11 | -0.09 | 0.16 |  |

Note. Due to missing data, N varies between 42 and 54. *P < 0.05.

Table S5 Spearman correlations of gender perception and gender role measures in the ASD group above the diagonal and the control group below.

|  | **Gender identity** | **Androgynous in childhood** | **Gender typicality** | **MF_M_** | **MF_F_** |
| --- | --- | --- | --- | --- | --- |
| **Gender identity** |  | 0.14 | 0.40^**^ | -0.11 | -0.02 |
| **Androgynous in childhood** | -0.09 |  | 0.08 | -0.01 | 0.06 |
| **Gender typicality** | 0.26 | 0.32^*^ |  | -0.10 | 0.06 |
| **MF_M_** | -0.15 | 0.12 | -0.30^*^ |  | -0.03 |
| **MF_F_** | -0.14 | -0.01 | 0.02 | -0.18 |  |

Note. Due to missing data N varies between 39 and 53. *P < 0.05.
