# Supplementary material for: Sexuality and Gender Role in Autism Spectrum Disorder: A Case Control Study
Source: PLoS One. 2014 Jan 31;9(1):e87961. doi: 10.1371/journal.pone.0087961 (PMC3909328; doi:10.1371/journal.pone.0087961)
Supplement: File S2 — Contains Tables S6–S11. (DOCX) [file pone.0087961.s002.docx]

**Table S6 Libido.** Have you been sexually aroused the past month?

| Libido | N (%) | | | |
| --- | --- | --- | --- | --- |
|  | Men | | Women | |
|  | ASD | Control | ASD | Control |
| No | 2 (7.7) | 0 (0) | 8 (34.8) | 1 (4) |
| 1-3 times | 4 (15.4) | 0 (0) | 5 (21.7) | 4 (16) |
| 4-6 times | 4 (15.4) | 1 (3.6) | 3 (13) | 5 (20) |
| 7-15 times | 6 (23.1) | 17 (60.7) | 4 (17.4) | 14 (56) |
| Every day | 10 (38.5) | 10 (35.7) | 3 (13) | 1 (4) |
| Total | 26 | 28 | 23 | 25 |

**Table S7 Sexual initiative.** Who takes initiative to have sex?

| Sexual initiative | N (%) | | | |
| --- | --- | --- | --- | --- |
|  | Men | | Women | |
|  | ASD | Control | ASD | Control |
| You | 2 (9.1) | 8 (28.6) | 2 (10.5) | 1 (4) |
| Both | 13 (59.1) | 20 (71.4) | 8 (42.1) | 22 (88) |
| Your partner | 4 (18.2) | 0 (0) | 8 (42.1) | 2 (8) |
| None | 3 (13.6) | 0 (0) | 1 (5.3) | 0 (0) |
| Total | 22 | 28 | 19 | 25 |

**Table S8 Sexual interest.** Are you interested in sex?

| Sexual interest | N (%) | | | |
| --- | --- | --- | --- | --- |
|  | Men | | Women | |
|  | ASD | Control | ASD | Control |
| Not at all | 0 | 0 | 3 (12.5) | 0 (0) |
| Not very much | 6 (23.1) | 3 (10.7) | 10 (41.7) | 3 (12) |
| Yes, quite | 11 (42.3) | 13 (46.4) | 6 (25) | 17 (68) |
| Very interested | 9 (34.6) | 12 (42.9) | 5 (20.8) | 5 (20) |
|  | 26 | 28 | 24 | 25 |

**Table S9 Orgasm frequency.** Have you had an orgasm during the past month?

| Orgasm frequency | N (%) | | | |
| --- | --- | --- | --- | --- |
|  | Men | | Women | |
|  | ASD | Control | ASD | Control |
| None | 1 (3.8) | 1 (3.6) | 10 (41.7) | 4 (16) |
| 1-3 | 8 (30.8) | 2 (7.1) | 6 (25) | 6 (24) |
| 4-6 | 4 (15.4) | 1 (3.6) | 2 (8.3) | 6 (24) |
| 7-15 | 10 (38.5) | 20 (71.4) | 6 (25) | 9 (36) |
| Every day | 3 (11.5) | 4 (14.3) | 0 (0) | 0 (0) |
|  | 26 | 28 | 24 | 25 |

**Table S10 Sexual orientation.** Whom are you attracted to?

| Sexual orientation | N (%) | | | |
| --- | --- | --- | --- | --- |
|  | Men | | Women | |
|  | ASD | Control | ASD | Control |
| None | 1 (3.85) | 0 (0) | 1 (4.2) | 0 (0) |
| Other sex | 22 (84.62) | 24 (85.71) | 9 (37.5) | 21 (84) |
| Both sexes | 1 (3.85) | 2 (7.14) | 13 (54.2) | 4 (16) |
| Same sex | 2 (7.69) | 2 (7.14) | 1 (4.2) | 0 (0) |
| Total | 26 | 28 | 24 | 25 |

Table S11 Spearman correlations of sexuality measures in the male ASD group above the diagonal and the female ASD group below.

|  | **Libido** | **Sexual initiative** | **Sexual interest** | **Orgasm frequency** |
| --- | --- | --- | --- | --- |
| **Libido** |  | -0.032 | 0.385 | 0.639^***^ |
| **Sexual initiative** | 0.367 |  | 0.132 | 0.056 |
| **Sexual interest** | 0.607^**^ | 0.685^***^ |  | 0.303 |
| **Orgasm frequency** | 0.713^***^ | 0.367 | 0.361 |  |

Note. Due to missing data N varies between 20 and 26. **P < 0.01, ***P < 0.001.
